# Managing Cancer Drug Resistance from the Perspective of Inflammation

**DOI:** 10.1155/2022/3426407

**Published:** 2022-09-19

**Authors:** Shuaijun Lu, Yang Li, Changling Zhu, Weihua Wang, Yuping Zhou

**Affiliations:** ^1^The Affiliated Hospital of Medical School, Ningbo University, Ningbo 315020, China; ^2^Institute of Digestive Disease of Ningbo University, Ningbo 315020, China; ^3^West China School of Basic Medical Sciences and Forensic Medicine, Sichuan University, Chengdu 610064, China; ^4^Ningbo First Hospital, Ningbo 315010, China

## Abstract

The development of multidrug resistance in cancer chemotherapy is a major obstacle to the effective treatment of human malignant tumors. Several epidemiological studies have demonstrated that inflammation is closely related to cancer and plays a key role in the development of both solid and liquid tumors. Therefore, targeting inflammation and the molecules involved in the inflammatory process may be a good strategy for treating drug-resistant tumors. In this review, we discuss the molecular mechanisms underlying inflammation in regulating anticancer drug resistance by modulating drug action and drug-mediated cell death pathways. Inflammation alters the effectiveness of drugs through modulation of the expression of multidrug efflux transporters (e.g., ABCG2, ABCB1, and ABCC1) and drug-metabolizing enzymes (e.g., CYP1A2 and CYP3A4). In addition, inflammation can protect cancer cells from drug-mediated cell death by regulating DNA damage repair, downstream adaptive response (e.g., apoptosis, autophagy, and oncogenic bypass signaling), and tumor microenvironment. Intriguingly, manipulating inflammation may affect drug resistance through various molecular mechanisms validated by *in vitro*/*in vivo* models. In this review, we aim to summarize the underlying molecular mechanisms that inflammation participates in cancer drug resistance and discuss the potential clinical strategies targeting inflammation to overcome drug resistance.

## 1. Introduction

Current cancer treatments (e.g., surgery, chemotherapy, radiotherapy, targeted therapy, and immune therapy) benefit an increasing number of patients who suffer from cancer [1]. Still, the effectiveness of these strategies is limited by drug resistance, which remains a primary obstacle to the curative treatment of various cancers [2]. Resistance to anticancer drugs can be divided into two categories: intrinsic and acquired drug resistance [3, 4]. Intrinsic resistance indicates that resistance regulators are present in a large number of tumor cells prior to chemotherapy, rendering treatment ineffective. Acquired resistance may develop during treatment as a result of nongenetic changes, which enhance tumor cell survival [5, 6]. The anticancer drug resistance mechanism involves many aspects, such as deregulated drug transport, altered target proteins/receptors, and abnormal regulation of cellular metabolic pathways [7, 8]. Of note, several new cancer treatment strategies have been applied to the clinic in response to the above mechanisms.

Inflammation is an immune response to bodily injury that can fight infection and trauma by removing harmful factors and damaged tissue, thereby repairing the tissue and returning it to normal [9]. In detail, in the early or acute phase of inflammation, pathogen-associated molecular patterns (PAMPs) are recognized by tissue macrophages or mast cells, which in turn activate the secretion of inflammatory cytokines, chemokines, vasoactive amines, and other substances, thereby enhancing the immune response around the blood vessels [10, 11]. Activated inflammatory cells produce anti-inflammatory cytokines as well as pro-inflammatory cytokines [12, 13]. The former mainly includes IL-4, IL-13, IL-10, and TGF-*β*, and the latter mainly includes TNF-*α*, IL-1*β*, IL-2, IL-6, IL-8, IL-17, and IFN-*γ*. Pro-inflammatory and anti-inflammatory cytokines interact to form a complex network whose dynamic balance determines the development and outcome of inflammation [14]. Studies have shown that multiple pathways are involved in the initial regulation of the inflammatory response, such as Jak/Stat signaling [15], NF-kB signaling [16], Wnt signaling [17], and Toll-like receptor signaling [18, 19]. Many similarities have been found between inflammatory response and tumor development in regulating signaling pathways and gene expression [20, 21]. For instance, NF-*κ*B is a transcription factor that can be activated by a variety of cytokines and plays a key role in inflammatory processes. In cancer, it is usually kept active and can regulate the cell cycle and apoptosis process by activating genes encoding related proteins, thereby inducing survival and promoting cancer progression [22].

**Figure 1:**
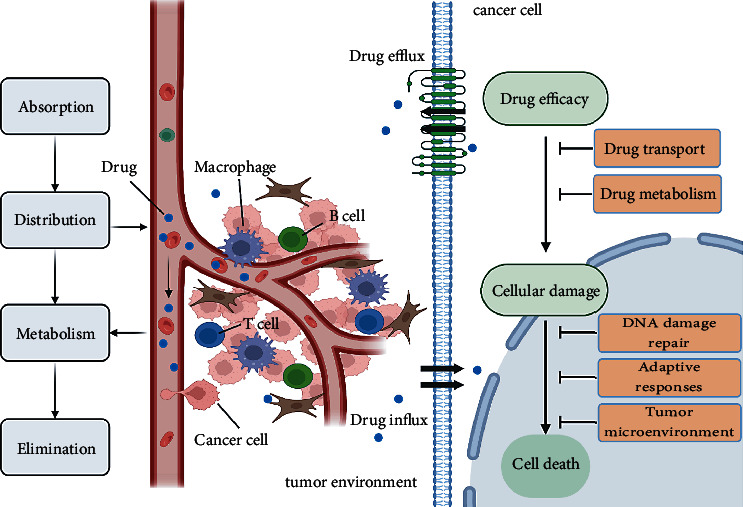
Factors affecting tumor drug resistance. Quantitative and qualitative changes in drug influx and efflux systems affect the distribution of anticancer drugs throughout the body and their accumulation in tumor cells. The drug metabolism of the body determines the efficacy of drugs. DNA damage repair, activation of pro-survival pathway or oncogenic bypass pathway, and changes in the tumor microenvironment are the main influencing factors of drug resistance.

As one of the hallmarks of cancer, multiline evidence indicates that inflammation contributes to tumorigenesis, tumor progression, metastasis, and resistance, yet its specific mechanism in most tumors remains unclear [23]. If the inflammatory response persists for too long, it may turn into chronic inflammation, which has many carcinogenic mechanisms, including inducing gene mutation, promoting angiogenesis, changing gene status, promoting cell proliferation and malignant transformation, etc [24]. It proved that inflammation plays a promoting role in the occurrence and development of tumors [25]. Nevertheless, there are few studies on the effect of inflammation on tumor drug resistance. In the current review, we suspect that inflammation plays a certain role in tumor drug resistance and focus on several aspects underlying inflammation-mediated drug resistance (Figure 1).

## 2. Inflammation-Mediated Changes in Drug Transport and Metabolism

Abnormal activation of drug efflux is one of the important reasons for tumor chemotherapy resistance. Meanwhile, the metabolic pathways are also major routes of drug resistance due to their drug-clearing and activation functions, particularly for anti-infectives and cancer drugs [26]. Abnormal activation of tumor efflux and excessive drug metabolism will cause the drug concentration in tumor cells to be lower than the killing concentration during tumor chemotherapy, resulting in tumor cell survival [27]. Therefore, exploring the activation level of drug efflux and drug metabolism in the inflammatory state is helpful to deeply understand the molecular mechanism of drug resistance under an inflammatory state. Here we attempt to explore the effects of inflammation on drug transport and metabolism.

### 2.1. Inflammation and Drug Efflux Transporters

There exist 48 ATP-binding cassette (ABC) transporters that can be subdivided into seven distinct subfamilies A-G [28, 29]. These drug efflux transporters reduce the intracellular drug concentration and inhibit drug efficacy [30]. Sufficient experimental results suggest that ABC transporter family proteins are associated with drug resistance, especially multidrug resistance protein 1 (MDR1; also known as P-glycoprotein and ABCB1), MDR-associated protein 1 (MRP1; also known as ABCC1), and breast cancer resistance protein (BCRP; also known as ABCG2) [31]. Some research has found that inflammation directly or indirectly impacts drug transporters. For example, peripheral inflammatory pain changes paracellular permeability and increases the expression and activity of P-glycoprotein (P-gp) at the blood-brain barrier, leading to arduous drug uptake by the brain [32]. MDR1 expression in immune cells has been studied in recent years. The inflammatory environment can cause upregulation of MDR1 in some cells involved in innate immunity [33]. Furthermore, the expression of BCRP was significantly reduced by IL-1, IL-6, and TNF-*α* during acute inflammation, and IL-2 can stimulate JAK3 activation, thus phosphorylating tyrosine residues in BCRP promote their drug efflux function [34]. Endotoxin-induced systemic inflammation in rats is correlated with the apparent changes in the placental expression of several drug transporters; with the increased level of pro-inflammatory cytokines IL-6 and TNF-*α*, the mRNA expression of Abcc3 increased, while the expression of transporters such as Abcb1a and Abcc2 decreased [35]. Researchers found that gene polymorphisms of ABCG2 potentially affect the serum levels of pro-inflammatory markers by drug efflux in some chronic inflammatory arterial diseases, implying an interaction between inflammation and drug transporters [36]. Several endogenous compounds related to inflammation development, such as cAMP and PGs, are also substrates of MRPs, which could provide new targets for the treatment of inflammatory diseases [37]. Together, inflammatory cytokines have different effects on drug transporters. Several cytokines (e.g., IL-6, TNF-*α*, and IL-1) inhibit drug efflux, while IL-2 increases drug efflux. The dual roles of cytokines indicate that the effect of inflammation on tumor cell drug resistance is the result of a multifactorial interaction, which is affected by the inflammatory microenvironment, the stage of inflammation induction, and the type of inflammation. Systematic studies on tumor resistance will help continue to elucidate the molecular events of inflammation-induced drug efflux and drug metabolism.

### 2.2. Inflammation and Drug Metabolism

In addition to drug efflux, activation or inactivation of drugs conducted by cytochrome P450 enzymes (CYPs) have been assumed to be important molecular mechanisms underlying cancer drug resistance [38]. The effects of cancer-induced inflammation on the hepatic metabolism of anticancer drugs have been noticed in recent years. Several inflammatory states were associated with decreased expression of some hepatic CYP enzymes such as 1A, 2A, 2C, 2E, and 3A subfamilies operated by pro-inflammatory cytokines at the transcriptional level [39]. Studies have shown that IL-6-mediated pathways, especially the MAPK/ERK and PI3K/AKT signaling pathways, are critical for the downregulation of CYP enzymes during inflammation [40], whereas IL-6 inhibitor tocilizumab can upregulate the expression of CYP3A4 mRNA [41]. Furthermore, JAK inhibitor has been found to reverse the IL-6-mediated downregulated CYP1A2 and CYP3A4 mRNA levels in HepaRG and PHHS cells, suggesting a prominent role of the JAK/STAT pathway mediated by IL-6 in CYP downregulation [42]. It is noteworthy that CYP enzyme activity is differentially affected by the presence of tumor-associated inflammation. For instance, increased activity of CYP2E1 was associated with raised serum levels of IL‐6, IL‐8, and TNF‐*α* [43]. Beyond this exception, IL-6 predominantly reduces CYP expression and thus attenuates the biotransformation of some drugs that are metabolized through CYP enzymes. Furthermore, studies found that after the TNF-*α* treatment for 24 h, the induction of CYP3A4 mRNA downregulation is not the same as protein downregulation. The latter is not obvious, suggesting that post-transcriptional mechanisms are involved in the downregulation of CYP protein levels or enzyme activity by TNF-*α* [44, 45]. As mentioned above, the same cytokines (e.g., IL-6 and TNF-*α*) had opposite effects on different P450 enzymes. Since different drug-metabolizing enzymes are selective for specific drugs, the characteristics of inflammation in patients during clinical drug treatment can indicate the activation or inhibition of specific drug enzymes, thereby assessing the risk of clinical drug resistance.

## 3. DNA Damage Response

The DNA damage response (DDR) is a protective mechanism under physiological conditions involved in DNA repair, checkpoint activation, and transcription regulation [46, 47]. On the other hand, it can help tumor cells survive the distractions of drug treatment [48–50]. Cancer treatment usually consists of some chemotherapy drugs that rely on the induction of DNA damage. Thus, the DNA damage repair capacity of cancer cells significantly influences the efficiency of DNA-damaging medications [51]. Recent studies indicate a positive correlation between IL-17 expression and the DDR in mice after cigarette smoke exposure, and excessive damage responses, in turn, lead to increased DNA mutation rates, which contribute to the genetic instability of cancer [52]. Meanwhile, by measuring DDR markers like activation of ataxia-telangiectasia mutated kinase (ATM), researchers found that inflammation in the gastric cardia mucosa may cause accumulated DNA damage, leading to mutagenesis and chromosomal rearrangements [53]. After treating lipopolysaccharides (LPS), the increased mRNA transcription of some DNA repair enzymes like AP endonuclease and DNA glycosylase excising *ε*A can be detected in rat intestines, which implies stimulation of the repair of oxidative DNA damage. Thus, inflammation may cause an imbalance in genome instability, more generally, to cancer development and drug resistance [54].

The DNA damage incurred during the inflammatory response triggers the activation of DNA repair pathways by stimulating a cascade of pro-inflammatory signals required for cell survival [55, 56]. Many DNA repair pathways are involved in regulating the transcription of molecules important for infection control, which implies a link between DDR and the inflammatory system [57, 58]. However, when DNA repair is not sufficient to deal with increasing DNA damage, the genes in the cells often mutate. Some cell mutations (e.g., BRAF V600E and EGFR T790M) can drive the development of tumors, causing tumor resistance. Among them, the occurrence of inflammation will accelerate this process [59]. For example, a recent study indicated the synergic effect between bacterial-driven inflammation and a mutant BRAF V600E to promote colon tumorigenesis in an enterotoxigenic *Bacteroides fragilis* (ETBF) murine model [60]. Similarly, the pro-inflammatory STAT3 pathway was found to be an important mechanism for EGFR T790M mutation-mediated drug resistance in NSCLC [61]. Inflammation can promote DNA repair in tumor cells, leading to tumor chemotherapy resistance. Blocking the inflammatory response is expected to act as an adjuvant therapy strategy for tumor-resistant treatment.

## 4. Downstream Adaptive Responses

**Figure 2:**
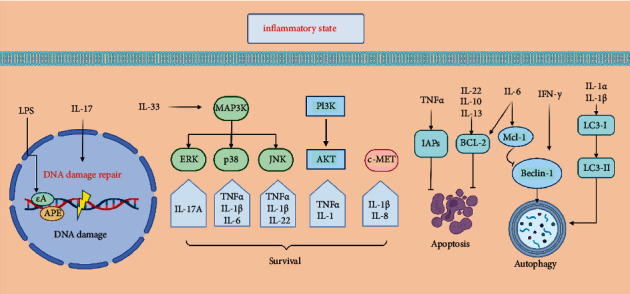
Molecular mechanisms of anticancer drug resistance. Inflammation not only aggravates DNA damage but also affects the expression of DNA repair enzymes, which leads to the instability of tumor genes and further promotes tumor drug resistance. Inflammation can regulate apoptosis and autophagy of tumor cells. For example, IL-6, 10, and 22 may inhibit the apoptosis of tumor cells by promoting the expression of BCL-2. Interferon and IL-1*α*/*β* can act on Beclin and LC3, respectively, to promote autophagy, while IL-6 can promote the binding of Mcl-1 and Beclin-1 to inhibit autophagy. Inflammatory mediators activate oncogenic bypass signaling pathways (e.g., MAPK, C-MET, or PI3K/AKT signaling pathways) leading to tumor resistance.

In addition to the intake and elimination of drugs, tumor cells can also obtain drug resistance through downstream adaptive responses [62, 63]. Currently, commonly used tumor therapy drugs are chemotherapeutic drugs and targeted therapeutic agents. Chemotherapy drugs ultimately lead to tumor cell apoptosis by injuring biomolecules within cells, while targeted therapies suppress tumor progression by inhibiting key regulators in tumor survival-maintaining mechanisms [64–66]. Tumor cells can escape death through various pathways, and these mechanisms are also important reasons for tumor drug resistance, where inflammation plays an essential role (Figure 2).

### 4.1. Evade from Apoptosis

Cancer cells can regulate the expression of some apoptosis-related proteins, such as B-cell lymphoma 2 (Bcl-2) and inhibitors of apoptosis proteins (IAPs), thus reducing their sensitivity to anticancer drugs that induce cell apoptosis and surviving the treatment [67, 68]. Bcl-2-related proteins are a large family that maintains the normal life state of cells by regulating apoptosis, which can be influenced by various cytokines associated with inflammation factors. For example, IL-13 has been found to induce Bcl-2 expression in airway epithelial cells [69, 70]. Results showed that the Bcl-2 level was increased after adding IL-6 in lymphoblast cells [71]. In addition, IL-22 activates the expression of the antiapoptotic protein Bcl-2 in renal cortex tissues [72]. Meanwhile, IL-10 has a role in protecting cells from apoptosis through upregulating Bcl-2 expression, especially in breast tumors [73]. It has worth noting that the effect of inflammation on apoptosis is not unilateral. By targeting the overexpressed Bcl-2 protein with the nanoparticle Bcl-2 inhibitor ABT-199, apoptosis of inflammatory cells and the reduction of IL-4 and IL-5 levels were observed [74]. Moreover, given that TNF*α* is one of the key mediators of cancer-related inflammation, at the same time, cIAP1 and cIAP2 function as key mediators of TNF*α*-induced the activation of NF-*κ*B and then allow the cell to survive against external interference, which indicates the interaction between inflammation and IAPs [75, 76]. Inflammation often accompanies the whole process of tumor occurrence and development and plays a key role in the process of tumor drug resistance [10]. Targeting tumor inflammation may act as a potential strategy to promote tumor apoptosis.

### 4.2. Autophagy

Autophagy is an evolutionarily conserved mechanism that disposes of excessive or potentially dangerous cytosolic entities through lysosomal degradation to maintain cellular biosynthesis and viability during stress conditions [77, 78]. Therefore, this process may result in the survival of cancer cells that are undergoing anticancer drug treatment. Inflammatory cytokines, including IFN-*γ*, TNF-*α*, IL-17, and the IL-1 family, are closely related to autophagy in tumors [79]. In human melanoma cells, blocking the IL-1 pathway by IL-1*α* or IL-1*β* treatment increases autophagy-related components such as LC3-I and LC3-II, indicating an increase of autophagy [80]. The overexpression of IFN-*γ* upregulated Beclin-1 mRNA expression and protein levels in the stomachs of mice, indicating that IFN-*γ* induces autophagy in part through upregulation of Beclin-1 [81]. However, inflammatory cytokines also play a role in inhibiting autophagy. For example, IL-6 overexpression was sufficient to block autophagy by supporting Beclin-1/Mcl-1 interaction and promoting arsenic-induced cell transformation in lung carcinoma cell lines [82]. Autophagy may also modulate inflammatory cytokine release and secretion through different mechanisms such as secretion of mediators of inflammation, regulation of inflammasomes, and p62/SQSTM1 proteins [83]. Since autophagy plays a dual role in tumor survival, the regulation of autophagy by inflammation is also context dependent. Further research is critically needed to understand the crosstalk between autophagic processes and inflammation as well as the underlying molecular mechanism.

### 4.3. Oncogenic Bypass Pathway

Activating the oncogenic bypass signaling pathways (e.g., MAPK, c-MET, or PI3K/AKT signaling) is crucial in tumor drug resistance. Studies have found that inflammation can directly or indirectly affect signal transduction in these pathways [84, 85]. For example, the JNK and p38 MAPK signaling pathways can be activated by pro-inflammatory cytokines such as TNF-*α* and IL-1*β*, associated with anticancer drug resistance in colon and liver cancer [86]. Studies have shown that the pro-inflammatory cytokine IL-22 can downregulate Cx43 gene transcription and promote keratinocyte proliferation and migration through the JNK-dependent pathway [87]. IL-17A stimulation leads to upregulation of Plet1 expression, which contributes to tissue regeneration and colonic tumorigenesis via regulating ERK [88]. IL-33 can activate the classical MyD88/IRAK/TRAF6 module, which activates three subfamily pathways of MAPK, including ERK, p38, and JNK [89]. Interestingly, activation of the JNK pathway can promote inflammatory actions such as directly activated NF-*κ*B by promoting I*κ*B*α* degradation, which indicates a positive feedback regulation of inflammation on the JNK pathway [90]. As for cholangiocarcinoma, inflammatory mediators such as IL-6 and TNF-*α* activate several pathways such as JAK-STAT, p38 MAPK, and Akt, resulting in increased cell growth and survival and proliferation [91]. Moreover, the expression of three major inflammatory cytokine genes, IL-1*β*, IL-8, and CXCL1, was positively correlated with c-MET expression in patients with brain metastases. In addition, the MAPK pathway is the central downstream transducer between c-Met and IL-1*β* [92].

Furthermore, under the chronic inflammation state, multiple inflammatory factors are associated with the PI3K/Akt pathway, which plays an important role in the initiation and development of downstream inflammatory pathways. For example, studies have shown that NF-*κ*B is triggered by PI3K/Akt by activating protein kinase C, which confirms the link between inflammation and oncogenic pathways [93]. In summary, inflammation promotes the activation of oncogenic bypass pathways in tumor cells, which may lead to targeted drug resistance. Therefore, the level of inflammation in the tumor microenvironment should be considered during clinical treatment, and combination with anti-inflammatory drugs may improve the efficacy.

## 5. Inflammatory Tumor Microenvironment

### 5.1. Oxidative Stress

Oxidative stress refers to the imbalance between the generation of free oxygen radicals and their elimination through the antioxidant defense system after being stimulated [94, 95]. As a by-product of normal metabolic processes, reactive oxygen species (ROS) are indispensable for various biological processes in normal and cancer cells [96]. Recent evidence suggests that ROS is closely related to tumor cell proliferation, heterogeneity, dormancy, and stemness, which are considered the critical requirements for tumor progression [97, 98]. However, the effects of excess ROS on tumor cells can be quite different in its anticancer properties, such as inducing apoptosis. Although most current chemotherapeutic agents increase ROS to cytotoxic levels when targeting cancer cells, exposure to ROS inevitably reduces the efficacy of chemotherapy in the long term, resulting in cancer drug resistance [99].

During inflammation, mast cells and white blood cells are recruited to the site of injury, resulting in increased release and accumulation of ROS at the site of injury due to increased oxygen uptake, leading to oxidative stress [100]. ROS promotes cancer growth and progression via different signaling pathways (PI3/Akt/mTOR, MAPK, etc.) when accumulating to a particular concentration [95]. Studies have shown that ROS-mediated drug resistance in cancer cells may be due to the activation of redox-sensitive transcription factors such as NF-*κ*B, Nrf2, c-Jun, and HIF-1*α* 30471641. Nrf2, a transcription factor involved in cellular homeostasis, is responsible for targeting genes related to cellular defense and plays a crucial role in regulating REDOX homeostasis [101]. Under oxidative stress, the specific cysteine residues of KEAP1 in the KEAP1-CUL3-RBX1 complex are destroyed, thus interfering with the ubiquitination process of Nrf2, which may ultimately lead to cancer drug tolerance [102]. Inhibition of Nrf2 gene expression has been found to reverse the resistance of head and neck tumors to iron death inducers [103].

Studies have found that oxidative stress and inflammation are interdependent and interrelated processes in the inflammatory RA joint, where inflammatory cells release large amounts of ROS, leading to excessive oxidative damage. In addition, many ROS and oxidative stress products enhance the pro-inflammatory response [104].

Exposure of phagocytes to pro-inflammatory cytokines such as TNF, IFN-*γ*, and/or IL-1*β* induces the formation of the NOX2 complex, which significantly increases intracellular ROS levels [105]. What's more, as a pro-inflammatory factor, TNF mainly acts through multiple TNF receptor-mediated signaling pathways, such as NF-KB and MAPKs, to regulate inflammatory responses. It is now clear that ROS/RNS are an integral part of TNF signaling because they are closely involved in numerous feedback loops, which are part of TNFR's downstream signaling pathways. Furthermore, ROS production also creates a positive feedback loop through autocrine TNF-*α*-mediated inflammatory cytokine/chemokine expression, which contributes to tumor progression [106, 107]. These findings suggest that inflammation may play a role in tumor resistance by stimulating the production of ROS.

### 5.2. Immune Response

**Figure 3:**
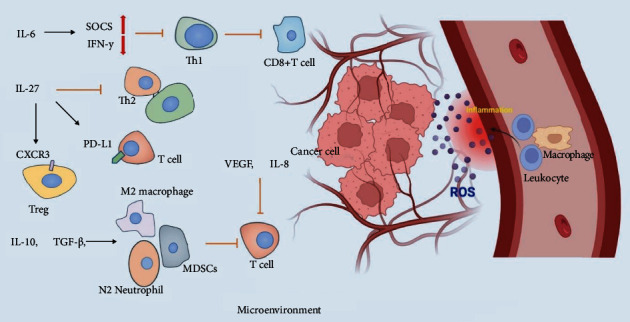
Inflammatory regulation of tumor microenvironment. In the tumor microenvironment, inflammatory factors can specifically activate or inhibit the function of immune cells, thus affecting the survivability of tumor cells. At the same time, the large amount of reactive oxygen species produced in the inflammatory site also plays a role in tumor resistance.

The immune system has a crucial role in cancer development and treatment [108, 109]. Deregulation of the immune response may cause cancer cells to evade immunogenic cancer cell death; however, the underlying molecular mechanisms involved in resistance to immunotherapy need further elucidation [109]. There is increasing evidence that immune response and systemic inflammation play an important role in tumor progression, and there is a complex interaction between them [110]. Researchers have found that tumor regulates the inflammatory environment by secreting soluble growth factors and chemokines so that inflammatory cells inhibit the anticancer T-cell response [111]. In the chronic inflammatory state, pro-inflammatory cytokines recruit immune cells such as M2, N2, and MDSCs, which are associated with suppressing immune surveillance and maintaining depleted T-cell phenotypes [112]. As a major pro-inflammatory cytokine, IL-6 plays an immunosuppressive role by inhibiting IFN-*γ* production and Th1 differentiation through SOCS1 induction, making CD8+T cells helpless. Studies have shown that upregulation of IL-6 activates STAT3, thereby inhibiting CD8 T-cell infiltration in non-small cell lung cancer and pancreatic cancer [113, 114]. While IL-10 has different effects on most immune cells and can inhibit the activation and effector functions of T cells, monocytes, and macrophages, it can also stimulate CD8+T cells and inhibit tumors to a certain extent [115]. The activation of MAPK signaling leads to increased VEGF and IL-8 expression, which then restrains T-cell recruitment and function [116]. Under the condition of inflammation, the metabolism of the stimulated immune cells may be disturbed, thus causing the abnormal function of the immune system [117]. Neutrophils under inflammatory conditions can affect other immune cells like T cells by producing chemokines and secreting granule contents and then promote immune paralysis of the adaptive immune system [118]. To avoid continuous inflammation, the cytokine IL-27 promotes Treg expression of T-bet and CXCR3 and triggers Tr1 cells. It has also been linked to increased expression of inhibitory receptors by T cells, antagonizing the development of Th2 and Th17 cell subsets. In addition, the observation that IL-27 induces PD-L1 and PD-1 suggests that IL-27 may be an important molecule in controlling cancer-related immune checkpoint mechanisms [119]. In summary, the relationship between tumor-associated inflammation and immune response still needs to be elucidated in many experiments. Furthermore, in-depth knowledge of the molecular mechanism of inflammation-influenced immune response could be beneficial to overcoming immunotherapy failure (Figure 3).

## 6. Targeting Inflammation to Overcome Drug Resistance

As mentioned in the previous discussion, inflammation can affect tumor development and drug resistance in multiple ways (Table 1). Of note, evidence increasingly suggests that drugs with anti-inflammatory properties have been shown to resist cancer. However, anti-inflammatory drugs are still in the early stages of clinical use in treating cancer due to drug risk. Here we summarize some of the current clinical advances that support the use of anti-inflammatory approaches for treating tumors (Table 2).

Conventional anti-inflammatory drugs like NSAIDs (aspirin) have been identified as a broad-spectrum anticancer agent based on data from epidemiological studies [129–131]. Aspirin can inhibit the nuclear translocation of NF-*κ*B, thereby inhibiting the PI3 kinase/Akt-mediated cell survival pathway and promoting cell apoptosis. In multiple myeloma cells, aspirin inhibited tumor cell proliferation and induced apoptosis by upregulating Bax and downregulating Bcl-2, changing the ratio of Bax/Bcl-2 [132]. Based on this, aspirin has been combined with other drugs in clinical trials treating drug-resistantnon-small cell cancers.

Several studies have shown that corticosteroids have significant anticancer effects both alone or in combination. As a selective COX-2 inhibitor and a nonsteroidal anti-inflammatory drug that inhibits prostaglandin production, celecoxib can induce apoptosis by activating transcriptional regulators of ER stress in hepatoma cells [133]. Dexamethasone can induce cell death in multiple myeloma mediated by miR-125b expression [134]. Other glucocorticoids, such as prednisone, its inhibition of prostate cancer growth may be due to inhibition of tumor-associated angiogenesis by reducing VEGF and IL-8 production [135].

As some infectious diseases that cause chronic inflammation have been linked to the development of cancer, anti-infective agents including antiviral, antibacterial, and antifungal drugs may play a role in cancer treatment [136–138]. Statins, which inhibit HMG-CoA reductase, also have anti-inflammatory properties and may have a beneficial effect on HCC caused by hepatitis [139].

Tumor immunotherapy is very effective in some patients, but resistance to which can also develop due to the immunosuppressive tumor microenvironment. Therefore, anti-inflammatory drugs that target immunosuppressive cells or cytokines may make tumor cells more sensitive to immunotherapy drugs and thus avoid resistance [115]. Studies found that monotherapy with COX-2 inhibitors or prostaglandin 2 (PGE2) receptor antagonists activate IFN-*γ*-driven transcriptional remodeling and synergy with immune checkpoint inhibitors to enhance effector T-cell accumulation in tumors [140].

As inflammatory mediators, cytokines and chemokines also mediate the tumor's response to external disturbances, which provides new targets for tumor drug therapy. For example, siltuximab and tocilizumab were used for ovarian cancer treatment as IL-6 antibodies [141]. MABp1 was used for colorectal cancer treatment as an anti-IL-1*α* antibody [142]. Blockade of inflammatory pathways such as TGF*β* signaling by galunisertib, which inhibits the TGF*β*R1 kinase, has been used to treat many types of cancer [143]. Due to the inflammation network within the tumor microenvironment being complex, inhibiting one molecule can cause a cascade effect, so the safety of the targeted drugs still needs to be tested in more trials.

Finally, some natural anti-inflammatory supplements might also help control cancer and they have the advantage of fewer side effects and greater safety. Berberine, which is a plant-derived alkaloid, may have the potential to improve colorectal adenomas [144]. Likewise, vitamin C has been extensively explored for potential anticancer effects [145].

Vitamin C's anti-inflammatory and antioxidant abilities can be attributed to its ability to regulate the DNA-binding activity of NF-*κ*B and reduce the production of inflammatory factors [146]. Dietary supplementation of vitamin C was found to result in a significant decrease in the mRNA expression of pro-inflammatory cytokines (e.g., IL-1*β*, IL-6, and IFN-*γ*) [147]. Cancer cells depend primarily on the gene expression of KRAS or BRAF to survive. Vitamin C gets inside these cancer cells and disrupts the expression of KRAS or BRAF genes. Once vitamin C has an effect, the mutation probability of cancer cells will also be greatly reduced, which reduces the resistance of targeted drugs [148].

The idea of targeting inflammation to treat drug-resistant tumors is innovative, but the molecular mechanisms behind the drugs still need extensive research and clinical trials to ensure the efficacy and safety of treatment regiments.

## 7. Conclusions

In this review, we summarize the recent studies on inflammation in influencing cancer drug resistance from several aspects including drug transport and metabolism, DNA damage response, downstream adaptive responses, oncogenic bypass signaling, and tumor microenvironment.

The activity of drug transporters and CYP enzymes changes in the inflammatory state [39]. Of note, different inflammatory cytokines have different effects on diverse drug transporters and CYP enzymes. Except for CYP2E1, the expression level of most liver drug enzymes is reduced in the inflammatory state, which inhibits the drug metabolism of tumors. In this regard, the mechanism of drug resistance to tumors remains to be studied. As DNA repair determines the potential of tumor cells to resist DNA-damaging drugs, the distinct roles of inflammatory cytokines in DDR emphasize the necessity to clarify the molecular mechanisms underlying the inflammation-related drug resistance. The role of multiple pro-inflammatory factors including IFN-*γ*, TNF-*α*, IL-17, and the IL-1 family in promoting drug resistance through autophagy has been confirmed by detecting the expression of autophagy-related molecules like LC3 and Beclin-1. It has also been suggested that IL-6, IL-22, and IL-10 can influence tumor drug resistance by regulating apoptosis-related proteins like BCL-2 and IAPs. Moreover, it is noteworthy that autophagy and apoptosis can also regulate inflammatory signaling pathways, thus forming a feedback regulatory pathway. We also summarized the regulatory role of some inflammatory factors in activating the oncogenic bypass signaling pathway, especially the cross-connections between NF-*κ*B and three typical pathways including MAPK, c-MET, and PI3K/AKT signaling. In the tumor microenvironment, the production of ROS is closely related to the failure of anticancer drugs. Some targets of the oxidative stress pathway are involved in the conduction of the inflammatory pathways, and some pro-inflammatory factors such as TNF*α*, IFN-*γ*, and IL-1*β* can promote the production of ROS. Although some studies have found that the function of some immune cells is suppressed under chronic inflammation, the specific mechanism of inflammatory components on cancer immune escape remains to be further elucidated due to the complexity of the immune system. At last, we summarize some anti-inflammatory applications in tumor therapy and propose some hypotheses for targeting inflammation against tumor resistance. Therefore, further studies on the molecular mechanism at multiple levels behind inflammation and cancer are required to overcome cancer drug resistance.

## Figures and Tables

**Table 1:** Summary of cytokine-mediated effects on drug resistance.

Cytokines	Downstream effector	Resistance mechanism	Role	References	Potential targeted drugs
IL-6	BCRP	Reducing drug efflux	Anti-resistant	[34]	Tocilizumab, sarilumab, clazakizumab, siltuximab [120]
	P-gp	Reducing drug efflux	Anti-resistant	[35]	
	CYP1A2 and CYP3A4	Inhibiting drug metabolism	Pro-resistant	[42]	
	CYP2E1	Promoting drug metabolism	Anti-resistant	[43]	
	Beclin-1/Mcl-1 interaction	Inhibiting autophagy	Anti-resistant	[82]	
	Bcl-2	Inhibiting apoptosis	Pro-resistant	[71]	
	JAK-STAT, p38 MAPK, Akt	Mediating survival signaling pathways	Pro-resistant	[91]	

TNF-*α*	BCRP	Reducing drug efflux	Anti-resistant	[34]	Adalimumab [121]
	P-gp	Reducing drug efflux	Anti-resistant	[35]	
	CYP2E1	Promoting drug metabolism	Anti-resistant	[43]	
	CYP3A4	Inhibiting drug metabolism	Pro-resistant	[44]	
	Beclin-1, LC3	Increasing autophagy	Pro-resistant	[79]	
	IAPs	Inhibiting apoptosis	Pro-resistant	[75]	
	JAK-STAT, p38 MAPK, Akt	Mediating survival signaling pathways	Pro-resistant	[91]	

IL-1	BCRP	Inhibiting drug efflux	Anti-resistant	[34]	Anakinra, canakinumab [122]
	LC3-I, LC3-II	Increasing autophagy	Pro-resistant	[80]	
	JNK, p38 MAPK, c-MET	Mediating survival signaling pathways	Pro-resistant	[86, 92]	

IL-22	Bcl-2	Inhibiting apoptosis	Pro-resistant	[72]	IL-22BP [123]
	JNK	Mediating survival signaling pathways	Pro-resistant	[87]	

IL‐8	CYP2E1	Promoting drug metabolism	Anti-resistant	[43]	Tocilizumab [124]
	c-MET	Mediating survival signaling pathways	Pro-resistant	[92]	

IL-17	DDR	Inhibiting DNA damage	Pro-resistant	[52]	Secukinumab, ixekizumab, brodalumab [125]
	ERK	Mediating survival signaling pathways	Pro-resistant	[88]	

IL-2	BCRP	Increasing drug efflux	Pro-resistant	[34]	Ro26-4550 [126]
IFN-*γ*	Beclin-1	Increasing autophagy	Pro-resistant	[81]	Emapalumab
IL-10	Bcl-2	Inhibiting apoptosis	Pro-resistant	[73]	AS101 [127]
IL-33	JNK, p38 MAPK, ERK	Mediating survival signaling pathways	Pro-resistant	[89]	N.A.
IL-13	Bcl-2	Inhibiting apoptosis	Pro-resistant	[69]	Suplatast tosilate [128]

**Table 2:** Summary of clinical trials of anti-inflammation drugs treating drug-resistant tumors.

Agent	Tolerated drug	Drug-resistant tumor type	Mechanism	Phase	Clinical trial number
Celecoxib	Platinum	Ovarian cancer/primary peritoneal cavity cancer	Stops the growth	II	NCT00084448
Dexamethasone	Platinum	Ovarian cancer	Stops the growth	II	NCT00003449
Aspirin	Osimertinib	“Non-small cell lung cancer”	Promotes cells apoptosis	I	NCT03532698
Prednisone	Hormone-resistant	Prostate cancer	Stops the growth	III	NCT00110214
Aspirin	EGFR-TKI	NSCLC	Promotes cells apoptosis	I	NCT03543683
Dexamethasone	Most MM drugs	Multiple myeloma	Inhibits tumor metastasis	II	NCT02626481
Dexamethasone	Chemotherapy	Recurrent plasma cell myeloma	Inhibits tumor metastasis	II	NCT03457142

## Data Availability

No data were used to support this study.
